# Characterization of adipose-derived stem cells of anatomical region from mice

**DOI:** 10.1186/1756-0500-7-552

**Published:** 2014-08-20

**Authors:** Arthur CL Luna, Maria EP Madeira, Thais O Conceição, José ALC Moreira, Rosa AN Laiso, Durvanei A Maria

**Affiliations:** Biochemistry and Biophysical Laboratory, Butantan Institute, 1500, Vital Brasil Avenue, Sao Paulo, Brazil; Medical School, University of Sao Paulo, 455, Doctor Arnaldo Avenue, Sao Paulo, 01246-903 Brazil

**Keywords:** Adipose tissue, Caspase-3, Cyclin D1, Multipotent cells, Mesenchymal stem cells, Mice

## Abstract

**Background:**

Stem cells constitute a group of great capacity for self-renewal, long-term viability, and multi-lineage potential. Several studies have provided evidence that adipose tissue represents an alternative source of stem cells, with the main benefit of adipose-derived stem cells being that they can be easily harvested from patients by a simple minimally invasive method and can be easily cultured. The aim of this study was to establish a culture protocol for obtaining and characterizing adipose-derived stem cells (ADSCs) from C57BL/6 J mice.

**Results:**

The results showed that the yield, viability, and cell morphology obtained differ according to the age of isolated anatomic regions of the adipose tissue from ovarian and epididymis. The results of determination of cyclin D1 showed uniformity in the expression between different populations of ADSCs. A significant increase in the expression of caspase-3 active, was also observed in large cell populations from mice after 120 days. ADSCs were positive for mesenchymal markers CD90 and CD105, Nanog, SSEA-1, CD106, and VEGFR-1, and negative for hematopoietic markers CD34 and CD45. A large number of cells in S + G2/M phases was also observed for both sexes, demonstrating high proliferative capacity of ADSCs.

**Conclusions:**

We observed that the adipose tissue of C57BL/6 J mice, isolated from the studied anatomic regions, is a promising source for obtaining pluripotent mesenchymal stem cells with high viability and proliferative response.

## Background

Stem cells constitute a group of great capacity for self-renewal, long-term viability, and multilineage potential [1]. The multilineage potential of embryonic and adult stem cells from bone marrow has been characterized extensively. In spite of the potential of embryonic stem cells for medical applications, many ethical and political issues accompany their use [2, 3]. Stem cells therapy and ex vivo gene delivery have provided two promising strategies for treatment of a vast array of inherited and acquired disorders. For these types of therapy, a reliable source of stem cells appears necessary. Researchers mainly work on two types of stem cells, embryonic and adult stem cells. However, immunorejection, tumorigenicity and ethical opposition have impeded the advancement of embryonic stem cells for clinical applications [4]. Compared with embryonic stem cells, autologous adult stem cells do not raise any major ethical or immunologic problems. Sources of adult stem cells include muscle, bone marrow, blood, epidermis, brain, liver, and, more recently, adipose tissue [3]. Stem cells derived from bone marrow were able to replace damaged heart muscle in mice after myocardial infarction [5].

However, the relatively low incident small tissue volume, difficult access, and disease-related malfunction of bone marrow-derived stem cells hamper their clinical usefulness. Several studies have provided evidence that adipose tissue represents an alternative source of stem cells and is routinely available in large quantities from low-risk techniques through non-invasive liposuction. Deposits of subcutaneous adipose tissue are an affordable, abundant, and highly capable replacement, thus providing a potential reservoir of adult stem cells in each individual. Many research groups, working independently, have demonstrated that adipose-derived stem cells (ADSCs) are able to differentiate in vitro into multiple lineages, among them adipocytes, chondrocytes, hepatocytes, osteoblasts, and endothelial, epithelial, hematopoietic, neuronal and myogenic cells [6]. Adipocytes derived from pluripotent cells that become precursors to mesenchymal are capable to turn into chondroblasts, osteoblasts, myoblasts or preadipocytes closely with vascular cells, stroma and extracellular matrix and are influenced by transcription factors and exogenous hormones. The components of extracellular matrix modulate the differentiation of preadipocytes and produce adhesive molecules which provide intercellular bonds and changes in morphology and size of adipocytes [7].

The main benefit of ADSCs is that they can be easily harvested from patients by a simple, minimally invasive method, and they can be easily cultured. Moreover, adipose-derived stromal/stem cells can be propagated more rapidly, and they retain their mesenchymal pluripotency, such as, 2.3 × 10^8^ cells to 120 mL, 80% confluent on the third pass [8]. Some investigations have indicated that ADSCs possess different surface epitopes and differentiation potential according to the localization of the fat pad from which the cells were derived. Epicardial adipose-derived cells tend to have a short population doubling time (45 ± 9.6 hours) than the epididymis adipose-derived stem cells (69 ± 16 hours). There are differences among deposits of ADSCs in different anatomic sites regarding their proliferative capacity and aging in vitro [9]. ADSCs from ovarian and epididymis have not yet been characterized. Therefore, the aim of this study was to establish a protocol for obtaining and characterization of ADCSs from male and female mice at different ages.

## Results

### Isolation and culture

**Figure 1:**
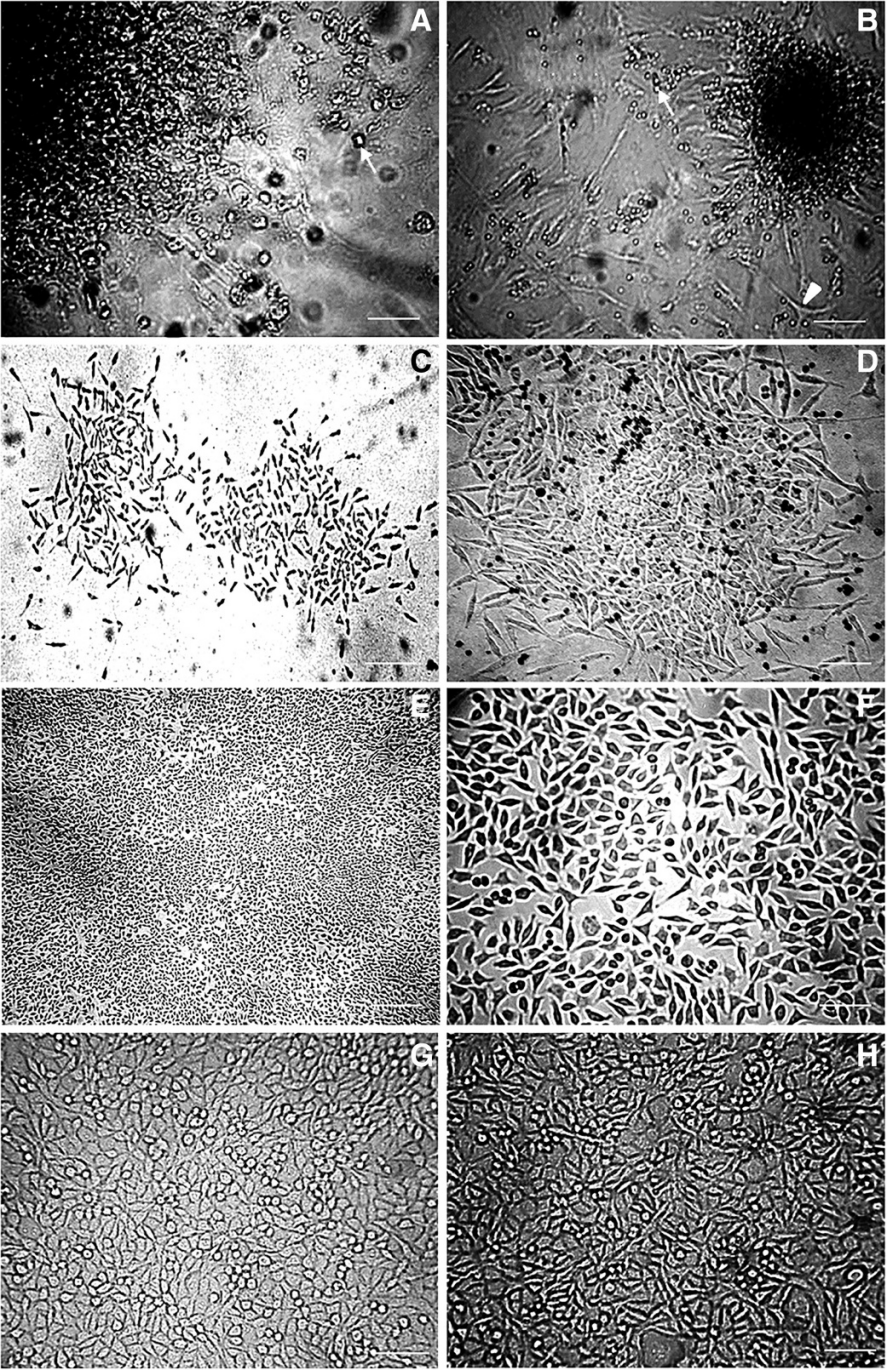
**Adipose-derived stem cells from C57BL/6 mice observed under inverted microscopy after culture.** For this study, were utilized 10 animals for sex, and experimental period of 30, 90, and 120 days (total of animals = 60). **A** and **B:** Initial explant of ADSCs (magnification, ×40 and × 20). It was observed in the cultures of adipose tissue, cells fusiform with appearance of fibroblast-like (arrowhead) and small cells adipose refractive rounded (arrow). **C** and **D:** Culture of ADSCs after 7 days (magnification, ×10 and × 20). **E** and **F:** Culture of ADSCs after 10 days. It is possible to observe cell confluence and homogenous population (magnification, ×5 and × 40). **G** and **H:** Culture of ADSCs above three passages on tissue culture plastic (magnification, ×20). Cell confluence was observed and homogenous population with small and lager cells in this period.

The samples of adipose tissue from ovarian and epididymis of different experimental groups were digested and cultured. During the initial days after plating, stem cells isolated from adipose tissue adhered to the surface of the culture plastic plates as a small cell population or polygonal spindle shaped forming a cells monolayer. The ADSCs proliferated rapidly in vitro, forming a homogeneous composition in monolayer, with fibroblast-like morphology (Figure 1). No difference was observed in fibroblast-like morphology presentation between large and small adipocyte obtained. The yield, morphology, and viability of cells obtained differed according to the age of tissue isolated from mice.

**Figure 2:**
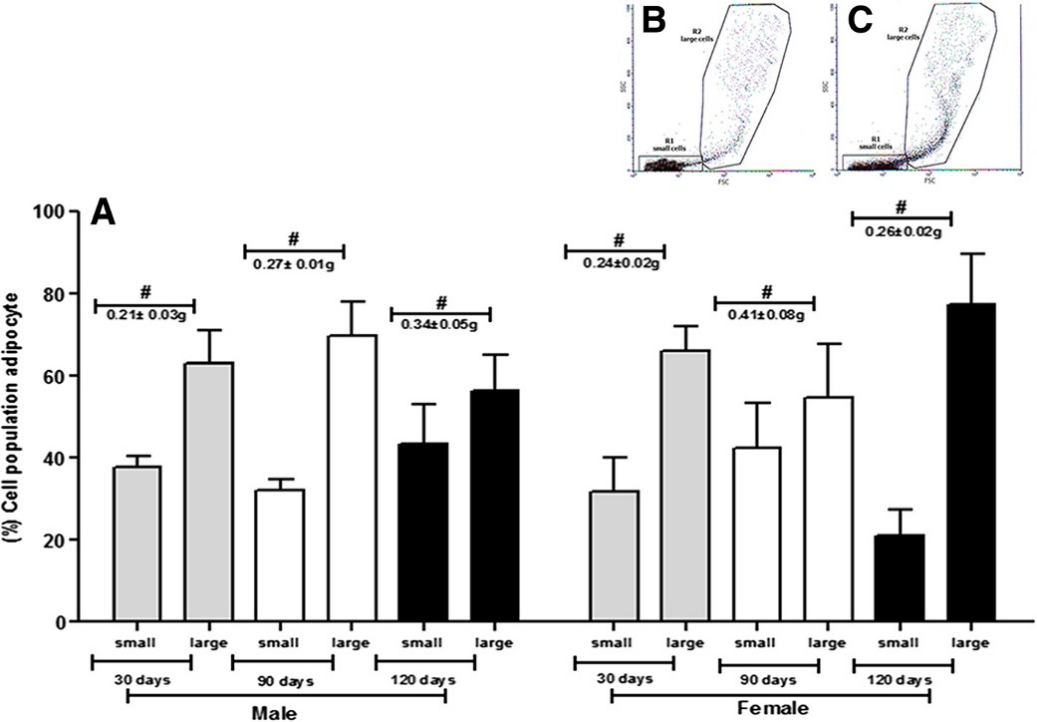
**Cell populations and weight of tissue (g) of ADSCs from C57BL/6 J mice males and females.** For this study were utilized 10 animals for sex, and experimental period of 30, 90 and 120 days (total of animals = 60). Results represent means ± S.D. from three independent experiments of each experimental group (days and sexes) in triplicate. **A:** The graph shows the cell yield acquired of ADSCs from C57BL/6 J mice males and females among 30, 90, and 120 days of age, subdivided into morphological group of small or large, after characterization by flow cytometry. Tissue weight (#). Statistical differences were obtained by analysis of variance ANOVA and Tukey-Kramer multiple-comparisons test. No significant difference was seen in the groups. **B** (Male 30 days) and **C** (Female 30 days): The scatter plot of FSC vs. SSC shows the subdivided into morphological group of small or large, after characterization by flow cytometry.

Male mice 30 days showed an average of 1.2 ± 0.2 × 10^5^ cells in 0.21 g of tissue while in the group of female mice of same age, the average obtained was 1.5 ± 0.9 × 10^5^ in 0.24 g of tissue. In male mice 90 days, the average was 1.6 ± 1.1 × 10^5^ cells in 0.27 g of tissue while females of same age had a mean of 1.4 ± 0.6 × 10^5^ cells in 0.41 g of tissue. At 120 days, there was a significant increase in yield of adipose tissue in relation to the total mass. In males 28.1 × 10^5^ ± 6.3 in 0.34 g of tissue was obtained and in females 34.3 ± 15.2 × 10^5^ cells in 0.26 g of tissue was obtained (Figure 2).

### Morphological characterization

The different populations of ADSCs were characterized by flow cytometry, as small or large, according to distinct morphological characteristics.

In males of 30 days 37.8% ± 2.5 of small cells was obtained and 62.9% ± 7.9 of large cells, in 90 days 32.2% ± 2.5 of small cells and 69.5% ± 8.5 of large cells and in 120 days 43.4% ± 9.7 of small cells and 56.4% ± 8.7 of large cells. On the other hand, in females of 30 days, 31.9% ± 8.2 was obtained and 66.0% ± 6.0 of small and large cells respectively, in 90 days 42.5% ± 10.8 of small cells and 54.7 ± 12.8% of large cells, and finally in 120 days, 21.2% ± 6.1% of small cells and 77.3% ± 12.2 large cells (Figure 2).

### Expression of stem cells markers on adipose cell populations by flow cytometry

**Figure 3:**
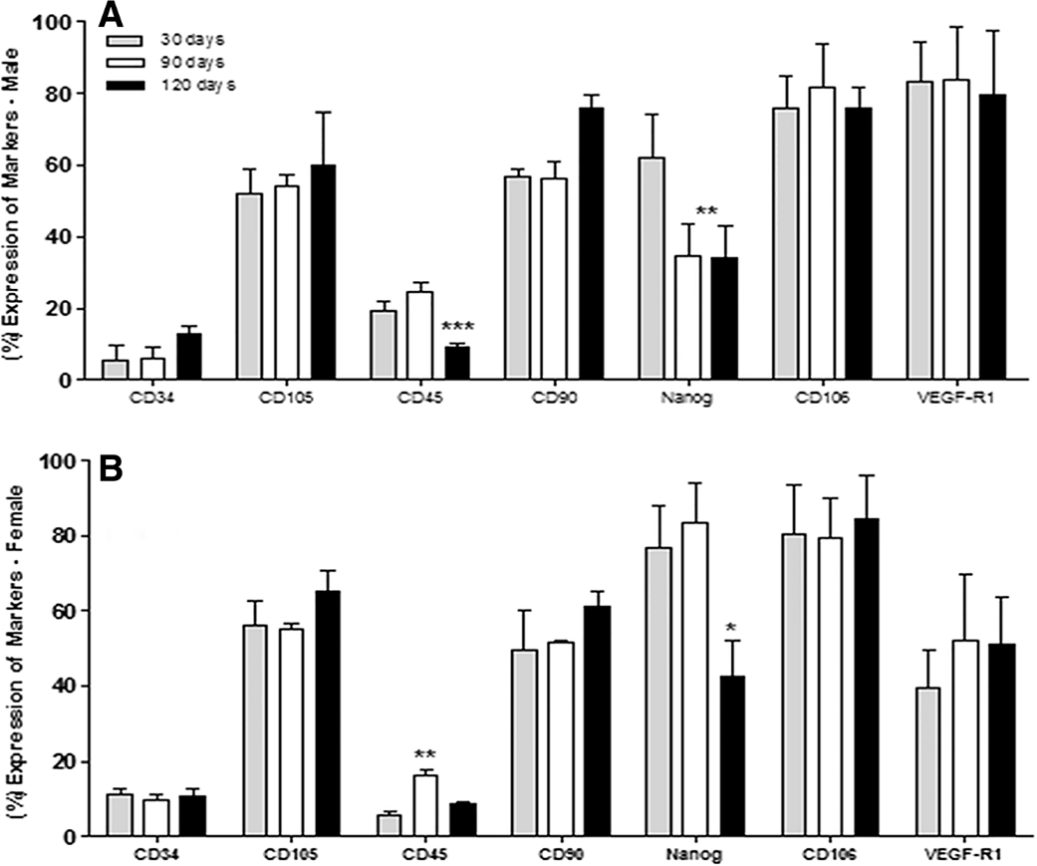
**Analysis of stem cell markers expression of ADSCs from C57BL/6 J mice males and females.** Results represent means ± S.D. from three independent experiments of each experimental group (days and sexes) in triplicate, obtained by flow cytometry analysis. The percentages of ADSCs mice males **(A)** and females **(B)** among 30, 90, and 120 days of age expressing hematopoietic stem cell markers (CD45 and 34), mesenchymal (CD90 and CD105), Nanog, CD106, and VEGFR-1 were correlated with the age and sex of animals. The difference between the positivity for expression of mesenchymal and hematopoietic markers was statistically significant. Statistical differences were obtained by analysis of variance ANOVA and Tukey-Kramer multiple-comparisons test. Statistical significance (p-value) *p < 0.05, **p < 0.01 and ***p < 0.001.

**Figure 4:**
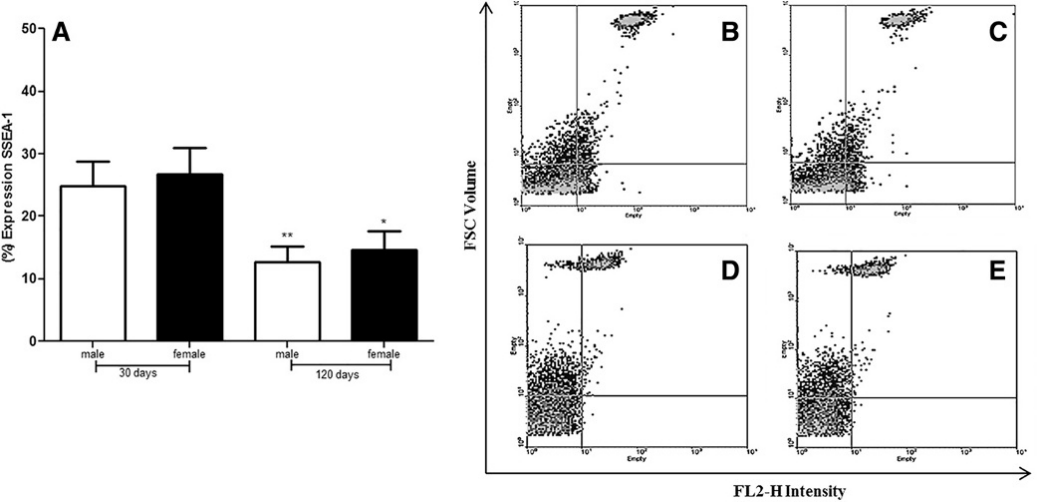
**Analysis of SSEA-1 expression of ADSCs from C57BL/6 J mice males and females.** Results represent means ± S.D. from three independent experiments of each experimental group (days and sexes) in triplicate, obtained by flow cytometry analysis. **A:** The graph shows ADSCs from mice males and females between 30 and 120 days of age characterized by flow cytometry compared with SSEA-1 expression. **B, C, D,** and **E:** A single fluorescent dye was used for a dot plot vs. FSC to visualize the expression of the markers of SSEA-1. **B:** SSEA-1 expression at female 30 days; **C:** SSEA-1 expression at female 120 days; **D:** SSEA-1 expression at male 30 days; **E:** SSEA-1 expression of male 120 days. Statistical differences were obtained by analysis of variance ANOVA and Tukey-Kramer multiple-comparisons test. Statistical significance (p-value) *p < 0.05 and **p < 0.01.

The expression of CD105, CD45, CD90, CD34, CD106, VEGFR-1, Nanog, and SSEA-1 was analyzed by flow cytometry. The results for males of 30 days were 52.2 ± 7.0%, 19.3 ± 2.6%, 56.8 ± 2.0%, 5.4 ± 4.4%, 75.7 ± 8.9%, 83,4 ± 10.7%, 62.0 ± 12.4% and 24.9 ± 3.9% respectively, for males of 90 days were 54.4 ± 3.0%, 24.5 ± 2.8%, 56.2 ± 4.7%, 6.2 ± 3.2%, 81.5 ± 12.1%, 83.9 ± 14.5% and 34.8 ± 8.7% respectively. The expression of SSEA-1 in 90 days is not represented in graphs, because there was no noticeable difference compared to the previous period in both sexes. For males in 120 days were 59.9 ± 14.9%, 9.4 ± 1.0%, 75.8 ± 3.7%, 12.7 ± 2.5%, 75.7 ± 5.9%, 79.3 ± 18.2%, 34.0 ± 9.1% and 12.7 ± 2.5%, respectively (Figures 3A and 4). In females, the expression in the group of 30 days was 56.2 ± 6.3%, 5.6 ± 0.9%, 49.7 ± 10.6%, 11.3 ± 1.5%, 80.4 ± 13.1%, 39.5 ± 10.1%, 77.0 ± 10.9% and 26.6 ± 4.2%. For the group of 90 days was 55.3 ± 1.2%, 16.2 ± 1.6%, 51.5 ± 4.2%, 9.7 ± 1.7%, 79.3 ± 10.9%, 52.1 ± 17.7% and 83.4 ± 17.8%, and females of 120 days, 65.1 ± 5.6%, 8.8 ± 0.5%, 61.1 ± 4.2%, 10.5 ± 2,1%, 84.3 ± 17.7%, 51.1 ± 12.7%,42.5 ± 9.7% and 14.7 ± 2.9%, respectively. The difference among the positivity for expression of mesenchymal and hematopoietic markers was significant (**p < 0.001) (Figures 3B and 4).

### Expression of cyclin D1 and caspase-3 active by flow cytometry

**Figure 5:**
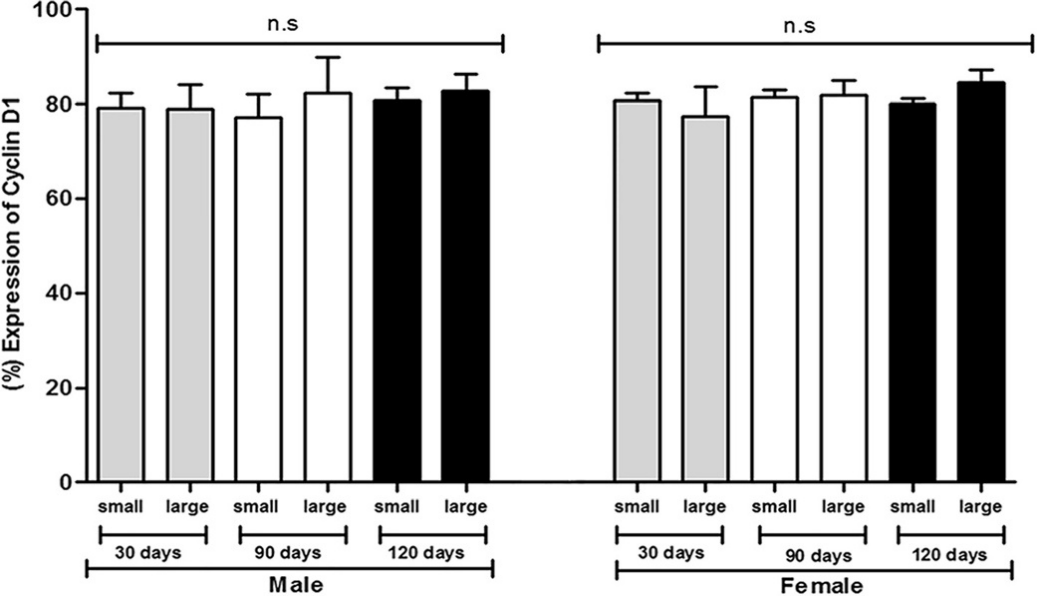
**Analysis of cyclin D1 expression of ADSCs from C57BL/6 J mice males and females** ***.*** Results represent means ± S.D. from three independent experiments of each experimental group (days and sexes) in triplicate, obtained by flow cytometry analysis. The graph shows ADSCs from mice males and females among 30, 90, and 120 days of age subdivided into morphologically large and small cells after characterization by flow cytometry compared with cyclin D1 expression. Statistical differences were obtained by analysis of variance ANOVA and Tukey-Kramer multiple-comparisons test. No significant (ns) difference at the groups.

The results of expression of cyclin D1 showed homogeneity increased among different populations of ADSCs and between small and large cells from males and females. Expression of cyclin D1 in males of 30 days was 85.0% for small cells and 84.2% for large cells; for the group of 90 days, it was 82.2% and 89.9% for small and large cells, respectively; and in the group of 120 days it was 83.5% for small cells and 86.4% for large cells. On the other hand, in females of 30 days, 82.3% and 83.7% was obtained for small cells and large cells, respectively, for the group of 90 days it was 82.9% for small cells and 84.9% for large cells, and finally for the group of 120 days, 81.2% of small cells and 87.3% of large cells (Figure 5).

**Figure 6:**
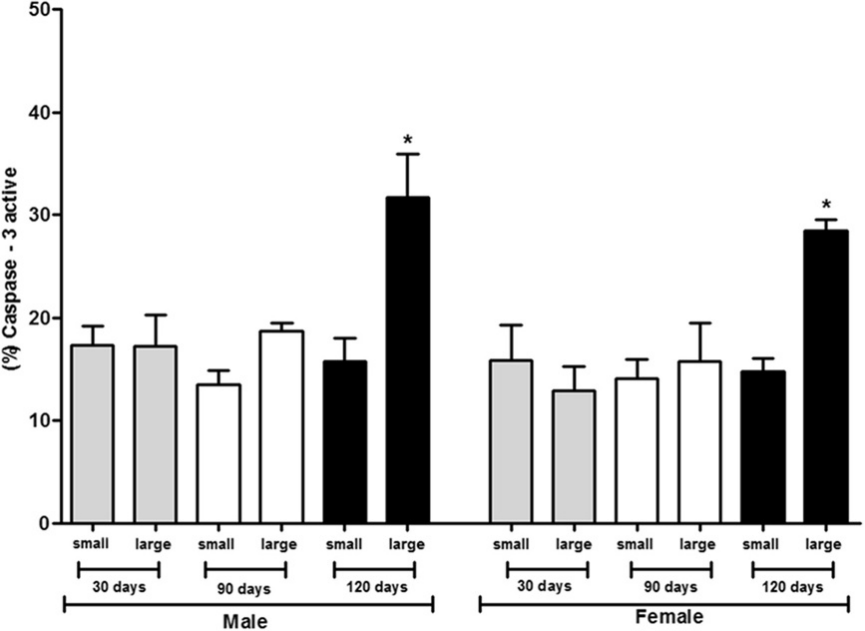
**Analysis of caspase-3 active expression from ADSCs from C57BL/6 J mice males and females** ***.*** Results represent means ± S.D. from three independent experiments of each experimental group (days and sexes) in triplicate, obtained by flow cytometry analysis. The graph shows ADSCs from mice males and females among 30, 90, and 120 days of age subdivided into morphologically large and small cells after characterization by flow cytometry compared with caspase-3 active expression. Statistical differences were obtained by analysis of variance ANOVA and Tukey-Kramer multiple-comparisons test. Statistical significance (p-value) *p < 0.05.

Expression of caspase-3 active for males of 30 days was 17.4% for small cells and 17.3% for large cells, for the group of 90 days, 13.5% for small cells and 18.7% for large cells, and for the group of 120 days, 15.7% and 31.7% for small cells and large cells, respectively. In females of 30 days, the expression was 15.8% for small cells and 12.9% for large cells, in 90 days it was 14.1% for small cells and 15.8% for large cells, and finally, in 120 days it was 14.7% for small cells and 28.5% for large cells (Figure 6).

### Cell cycle phases distribution

**Figure 7:**
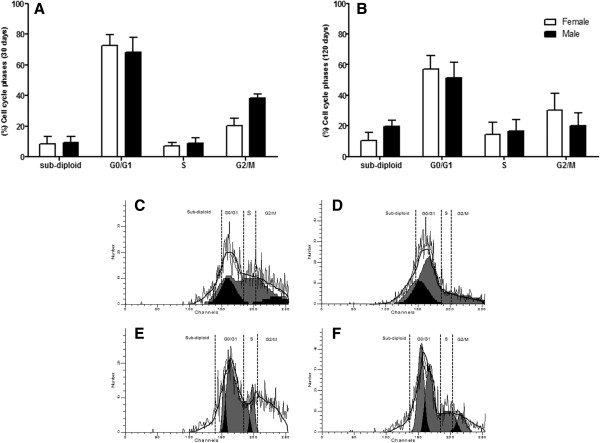
**Analysis of cell cycle phases of ADSCs from males and females.** Results represent means ± S.D. from three independent experiments of each experimental group (days and sexes) in triplicate, obtained by flow cytometry analysis. **A:** The proportion of ADSCs from C57BL/6 J females and males 30 days residing in sub-haploid, G0/G1, S and G2/M are indicated; **B:** The proportion of ADSCs from C57BL/6 J females and males with 120 days in sub-haploid phase, G0/G1, S and G2/M was indicated. Statistical differences were obtained by analysis of variance ANOVA and Tukey-Kramer multiple-comparisons test. Statistical significance (p-value) *p < 0.05. **C**, **D**, **E**, and **F**: Cell cycle analysis of flow cytometric DNA histograms. **C:** Histogram shows the proportion of ADSCs from C57BL/6 J females 30 days in sub-haploid phase, G0/G1, S and G2/M cell cycle phases; **D:** Histogram shows the proportion of ADSCs from C57BL/6 J males 30 days in sub-haploid phase, G0/G1, S and G2/M cell cycle phases; **E:** Histogram shows the proportion of ADSCs from C57BL/6 J females 120 days in sub-haploid phase, G0/G1, S and G2/M cell cycle phases; **F:** Histogram shows the proportion of ADSCs from C57BL/6 J males 120 days in sub-haploid phase, G0/G1, and G2/M cell cycle phases. The histograms were obtained by ModFit 3.2 software.

At 30 days, accumulated cells in G0/G1 phase were observed, in which a large number of cells were found, being 68.3% for males and 72.4% to females. At 120 days, there was a decrease of cells in this phase, being 51.3% for males and 57.1% for females. In the synthesis phase, there was an increased number of cells. Regarding S/G2/M phases, there was a higher cell concentration for males at 30 days, with 47.5% and for females 27.6%. At 120 days the results for the same phases were inversely proportional for both sexes, compared to the previous period. While in the sub-haploid population in 30 days, there was a similar proportion for both sexes, with 9.2% for males and 8.5% for females. However, in 120 days there was an increase in the number of cells in the sub-haploid (Figure 7).

## Discussion

Research shows that human adipose tissue obtained from liposuction is an abundant and an available source of ADSCs and can be used for research purposes. Stem cells from adipose tissue were first isolated from collected fragments of rodent adipose tissue through open surgery. Later, several groups have reported the isolation of human ADSCs taking advantage of liposuction aspirate as a tissue source [9]. The ADSCs cells display a reproducible and consistent phenotype based on cell-yield viability, adipocyte differentiation, and cell-surface marker phenotype. In this work, the use of mechanical and enzymatic techniques to isolate ADSCs from mice, proven to be effective once an adherent single cells layer were obtained, acquired a fibroblast-like morphology, this being one characteristic of mesenchymal stem cells. The characterizations of fibroblast-like morphology have been used as criteria in assessing the potential difference of stem cells of mesenchymal origin [10, 11].

In this study, during the first days after culture, ADSCs adhered to the flask surface, as a small or fusiform population of polygonal cells. The ADSCs expand easily in vitro and exhibited a homogeneous fibroblast-like morphology. The cells were sub-cultured within 1–2 days, when cells became 90% confluent.

The yield of ADSCs was expressive with an average of 87.0%, and cell viability greater than 94.0%. The data showed that the number of ADSCs and viability acquired from mice at different ages was greater than that obtained in recent studies. These studies showed that 1 g of adipose tissue can yield approximately 4-5×10^6^ cells, including 1×10^6^ adipocytes, 1×10^6^ ADSCs, 1×10^6^ vascular endothelial cells, and 1x10^6^ other cells. [12, 13]. Our group obtained recently stem cells of synovial membrane from equines and the data corroborate in ADSCs yield (data not shown). When comparing the results with the number of cells that can be obtained from human adipose tissue, acquired by liposuction, there were higher mean values in this study. About 2.0% of human lipoaspirate are ADSCs, or in 10 g of adipose tissue, about 0.2 g of mesenchymal stem cells could be isolated and used to perform cell therapy because these cells possess a large capacity to expand in culture, even accounting for a small fraction of adipose tissue [14]. The isolation of adipose tissue from infundibulum of ovarian, epididymis, and proximal regions showed significant cell yield compared to other studies [15–17] and satisfactory viability when compared with results obtained in humans, which was about 93.9 ± 3.3% based on test trypan blue exclusion [15].

The weight of tissue and amount of isolated cells differed according to the age and sex of animals. It was observed that females 90 days showed higher accumulation of adipose tissue in studied regions compared with males of the same age, however, at 30 and 120 days, the males showed a higher amount of adipose tissue than females. Comparing the cell yield with sex and age of animals, it was observed that there was a significant increase in relation to age, represented mainly by large cell populations. However, there was no relation among the weight of adipose tissue, the amount of cells obtained, cell morphology, and age of animals.

The amount and anatomical region of stored fat differs in females and males. Females tend to have a higher body fat content, localized subcutaneously, while males have less total body fat and their adipose tissue predominates in the visceral region. Estrogen is a major factor involved in this sexual dimorphism as it promotes subcutaneous fat accumulation, has anti-inflammatory properties, and is a strong regulator of appetite and energy expenditure [18]. Estrogen regulates the amount of white adipose tissue (WAT) in females, but its role in males and whether WAT effects involve expression of estrogen receptor-alpha (ER-α) or receptor-beta (ER-β) were as yet unclear [19, 20].

Mesenchymal stem cells (MSCs) have no expression for hematopoietic surface markers CD34, CD45, and CD14 and endothelial cells CD31, and are positive for STRO-1, CD29, CD73, CD90, CD105, CD166, and CD44 [21]. The data obtained in this study showed that the cells studied exhibit significant expression of mesenchymal markers CD90 and CD105, corroborating other results [22], and low expression of hematopoietic markers CD34 and CD45. Thus, the results suggest that mesenchymal stem cells were isolated from adipose tissue of mice.

The expression of CD105 and CD34 increased significantly with enhanced VEGF expression [23]. Expression of markers CD9, CD29, CD44, CD49d, and CD106 interfere with the proliferation and differentiation of hematopoietic stem cells. Preliminary studies indicated that adipose tissue-derived stromal cells are capable of supporting hematopoiesis in vitro [24].

The markers did not differ in relation to the sex of animals. However, there was a higher positivity of markers in large-cell populations, as well as the findings of other authors [25], who observed that large cells showed higher expression of markers of mesenchymal cells. It was observed that at mice 120 days there was a decrease in the expression of markers of mesenchymal cells in both sexes and a not very significant increase in expression of hematopoietic markers. The increase of these markers may be explained at this age for a period of maturation of ADSCs, causing the greatest potential for hematopoietic cells.

According to reports in the literature about stem cells derived from stromal vascular fraction (SVF) of adipose tissue, up to three passage, has distinct populations of cells divided into small and large stem cells, supporting results obtained from ovarian and epididymis. Following adherence of the SVF to tissue culture flasks (MSCs, passage 1) the immunophenotype became more homogenous for both the large and small cells [25]. In literature, 53.0% of mesenchymal large cells exhibit 79.0% viability; whereas 47.0% of small cells have 94.0% viability, thus, the size of mesenchymal cells interferes with cell viability [25]. In our study, were observed the existence a significant increase in the expression of caspase-3 active at 120 days, in large cells populations, demonstrating that during this period the activation of apoptosis occurs, which may be a balance mechanism of the cell number when they reach maximum potential of maturation. Regarding the expression of cyclin D1, no significant difference was seen among markings in populations of ADSCs and between sexes.

Researchers described that the age is a limiting factor for the use of adult stem cells due to accumulation of intrinsic events, such as DNA mutations, as well as the extrinsic (changes in the stem cell niche). It is assumed that, with advancing age, the mechanism responsible for suppression of development of tumors, such as senescence and apoptosis, may induce a decline of multiplicative function of stem cells [26]. Various intrinsic programs and pathways facilitate the unique characteristics of stem cells, and these programs, in charge of everything maintenance, to proliferation, to eventual differentiation of the stem cells, have to be tightly controlled by the microenvironment [27].

In early G1-phase, cyclin-D family comes together in a holoenzyme complex with one or two catalytic subunits of cyclin-dependent kinases (CDKS), Cdk4 e Cdk6 [28]. Analysis of cell cycle phases that the age represents, for both sexes, showed that the major cell populations are in G0/G1, corroborating the expression of the cyclin-D1. However, in 120 days there was a decrease in the phases G0/G1 for both sexes. A large number of cells were observed in phases S + G2/M, for both sexes, demonstrating high proliferative capacity of the ADSCs, as we discussed previously. However, in 120 days an increase of cell number was observed in phase sub-haploid for both sexes. This increase demonstrates the loss of cell viability, also evidenced by the significant increase of the caspase-3 in the same period. Most cells with sub-haploid DNA content correspond to apoptotic cells with fragmented DNA or condensed chromatin [29]. This process may result in the presence of cellular debris and nucleus containing small amounts of DNA [30]. Then, the fraction sub-haploid quantified in flow cytometry may contain, in addition to apoptotic cells, cellular and nuclear fragments, nucleus or micronucleus with normal content of DNA, but containing a different chromatin structure [31].

In early embryonic development, cell fate is regulated by the core transcription factor network, which includes octamer-binding transcription factor 4 (Oct-4), sex determining region Y-box2 (Sox-2), homeobox protein Nanog, signal transducer and activator of transcription 3 (Stat-3), and some other factors [32]. Nanog is the first transcription factor that is restricted to the inner cell mass after compression [33]. Nanog represses embryonic ectoderm differentiation but has little effect on other lineages, whereas Sox-2 and Sox-3 are redundant and repress mesendoderm differentiation [34]. Nanog has been detected only in proliferating cells, but not in MSCs induced to differentiate. The percentage of cells expressing Nanog was maintained throughout early passages of MSCs, but then started to decrease in late passages in MSCs from adipose tissue and heart but not from bone marrow [35]. We found that Nanog expression was positive for both sexes and ages. Expression of Nanog for females of 90 days increased significantly compared with males of the same age. At the pre-implantation stage, murine embryos, human germ cells, and teratocarcinoma stem cells express certain molecular receptors known as Stage Specific Embryonic Antigens (SSEA) on their membrane surface [36]. SSEA-1 and Nanog occur in embryonic stem cells; both have an important role in maintaining the capacity for self-renewal [37]. Thus these markers have been used to characterize mouse and human embryonic stem cells [38]. The expression of SSEA-1 in the cells supports the marking of Nanog, being positive in both sexes and ages analyzed, but there was no significant difference between sexes. Cell culture at 90 days is not represented in the graphs because there was no noticeable difference compared to the previous period. There was a significant decrease in SSEA-1 expression for both sexes. These results indicate that these cells are pluripotent and the method for establishing them is feasible. The literature showed in histological and cytometric analyzes that SSEA-1 was expressed at high levels in ADSCs. In ADSCs cultured, at passage 0, it was expressed at a higher level than CD34 [39]. Gender differences were found to affect the osteogenic capacity of ADSCs, with ADSCs from males differentiating more rapidly and more effectively than ADSCs from females in vitro; the adipogenic potential was unchanged irrespective of age, while the osteogenic potential appears to decrease with increasing age. These differences are likely due to the different steroid functions in males and females with hormone levels ranging at different phases of life [35].

Members of the VEGF family show different affinities for one of three VEGF tyrosine kinase receptors: VEGF R-1, R2, or R3 [40]. Studies targeting genes in mice have shown the importance of VEGFs and VEGFRs in the development of the vascular system [41]. Despite a high degree of homology within the kinase domains, the signaling properties of different VEGFs greatly differ. The fact that VEGFR-1be is usually expressed at low levels has hampered progress in elucidating its signal transduction pathways. VEGFR-1 has been shown to mediate monocyte migration, recruitment of endothelial cell progenitors, hematopoietic stem cell survival, and release of growth factors from liver endothelial cells [42, 43]. VEGFR-1 is a positive regulator of monocyte and macrophage migration, and has been described as a positive and negative regulator of VEGFR-2 signaling capacity. Moreover, the migration of hematopoietic progenitor cells, as well as monocytes, towards a gradient of VEGF is mediated through VEGFR-1 [41]. The results of the determination of VEGFR-1 showed uniformity in the expression between 30 and 120 days of age, however, receptor expression in females is significantly decreased compared with males of the same age. The high VEGFR-1 expression can be explained by the change in adipose tissue because it is one of the most vascularized tissues of the body, and a close functional relationship exists between fat tissue and its vasculature.

Other studies have reported the involvement of VEGFR-1 signaling in fat tissue formation. For example, mice deficient of placenta-derived growth factor (PlGF, specific ligand for VEGFR-1) have lower body weights during the later stages of diet-induced obesity. However, pharmacologic inhibition of PlGF had no apparent effect and adipogenesis is dependent on VEGF-mediated formation of new blood vessels [44]. MSCs can also increase angiogenesis. Rats treated with MSCs had significant increases in numbers of newly formed capillaries at the boundary of the ischemic lesion in rats treated with human MSCs. Decreased VEGFR-1 expression in females when compared to males may be related to the pregnancy period. In rats, mRNA for VEGF_164_ is increased during pregnancy (5.0 fold increase on 12th day) and during lactation (18.5-fold increase on 7th day). VEGF _120_, _165_ and _188_ amino acid isoforms are expressed during pregnancy, lactation, and involution, and their abundance relative to one another does not alter during the mammary gland cycle. In mice, there is an increase in VEGF mRNA levels during the phase of lactation (maximal 9.7-fold at 7th day); levels of VEGF decreased progressively during the phase of involution. In relation to VEGFR-1 and VEGFR-2, a quantitative analysis in the rat mammary gland revealed an increase in VEGFR-2 during pregnancy (1.6- fold at 4th day). During lactation, VEGFR-1 (2.7- fold at 7th day) and VEGFR-2 (3.8- fold at 7 ^th^ day) increased [45].

The reproducibility and consistency of these primary human cells support their value as an adult stem cell model, and may be used with great potential in basic research and preclinical studies. Several studies have demonstrated the potential of these stem cells. ADSCs were able to improve peripheral nerve regeneration in vivo when compared with no conduits cells [46]. The ability to isolate a consistently homogeneous population of undifferentiated adult stem cells from adipose tissue from studied anatomical regions, supports their potential utility in future tissue-engineering applications. Adipose tissue represents a precious source of multipotent cells with a great potential for several clinical applications, above all in the field of regenerative medicine [11]. ADSCs have the relative advantages of accessibility and abundance. This means that there is less pain to the patient in obtaining the cells and it is easy to obtain a sufficient quantity of cells.

## Conclusion

The use of mechanical and enzymatic techniques to isolate ADSCs of mice is proven to be an effective method, once an adherent single cells layer was obtained. The data showed that number of ADSCs, and viability, acquired from adipose tissue of mice, at different ages and different anatomical sites, is greater than that obtained in recent studies. The high VEGFR-1 expression can be explained by must be change the adipose tissue because it is one of the most vascularized tissues of the body and a close functional relationship exists between fat tissue and its vasculature. The data obtained in this study showed that cells studied exhibit significant expression of mesenchymal markers CD90 and CD105 and pluripotency markers Nanog, SSEA-1, as well as a marker of proliferation potential Nanog. These results indicate these cells are pluripotent and that the method for establishing them from ADSCs is feasible. The results suggest that adipose tissue, isolated from the studied regions is a promising alternative source of stem cells. The yield and cell viability of the ADSCs acquired in this study make these cells a potential source in tissue engineering applications.

## Methods

### Animals

All experiments protocols were approved by ethics committee on animal use from Butantan Institute. For this study, 60 animals were used. Adipose tissue was collected from normal mice C57BL/6 J, males and females of 10 mice/group (aged 4 to 16 weeks old), weighing between 20–25 g**,** from Central Bioterium of Butantan Institute, São Paulo, Brazil. The animals were sacrificed in accordance with the standards described in Use of Experimental Animals at Johns Hopkins - Guide to the Care and Use of Experimental Animals.

### Isolation and culture of ADSCs

In sterile conditions, under laminar flow, the abdominal cavity was exposed and adipose tissue was isolated from regions close to the ovarian infundibulum in females and in the lateral epididymis region in males. The tissue was mechanically separated and the material was processed under laminar flow also in such a way as to be eliminated adipose tissue subjacent, leaving the dermo-adipocyte sheet impregnated in Hank’s Balanced Salt Solution (HBSS) (Gibco, Carlsbad, CA, United States). The small fragments on a Petri plate of 35 mm were incubated with type IV collagenase 0.2 U/ml for 3 h at 37°C in 5% CO_2_. Then, the collagenase was neutralized with 10 ml of fetal bovine serum (Sigma-Aldrich, St. Louis, MO, United States), the cellular debris were removed and remaining material, consisting of vascular cells, fibroblasts and adipocytes was solubilized in HBSS and filtered through a 40 μm membrane. The concentrate obtained from the previous step was centrifuged for 10 minutes at 274 × g and at 4°C, the supernatant formed was aspirated, the cell pellet resuspended in 1 ml of complete culture medium and cell culture flats were incubated containing the material. After growth and expansion, to subconfluence, the cells were resuspended in culture medium, trypsinized and filtered. The filtrate was collected on cell culture flasks containing 50 ml of saline solution supplemented with antibiotics. Finally, cells were quantified on Malassez chamber, the concentration adjusted to 2 × 10^5^ ml and plated for different analyzes. ADSCs above three passages on tissue culture plastic (P3) were analyzed for surface marker expression.

### Characterization of ADSCs by flow cytometry

After three passage ADSCs were detached using trypsin-EDTA 0.2% (Gibco, Carlsbad, CA, United States) and were counted. Then, 3% rat serum was added to aliquots of about 2 × 10^5^ cells in centrifuge tubes. The cells were incubated on ice for 30 min, resuspended in PBS and pelleted by centrifugation for 10 min at 274 × g. Then, the cells were stained with CD105 (AbD Serotec, Raleigh, NC, United States), CD45 (Sigma-Aldrich, St. Louis, MO, United States), CD90 (Abcam, Cambridge, MA, United States), CD34 (Santa Cruz Biotechnology, Dallas, TX, United States), CD106 (Becton Dickinson, San Jose, CA, United States), VEGFR-1 (Sigma-Aldrich, St. Louis, MO, United States), Nanog (Abcam, Cambridge, MA, United States) and SSEA-1 (Abcam, Cambridge, MA, United States) at a concentration of 1 μg/ml at 4°C for 30 min. The corresponding isotope antibody was used as negative control and as a secondary antibody was used Goat anti Mouse IgG (H/L): FITC (AbD Serotec, Raleigh, NC, United States). The cells were pelleted, washed twice with PBS and fixed with 1% paraformaldehyde. Then, fluorescence-activated cell sorting (FACS) analysis was performed on BD Biosciences FacsCalibur flow cytometer (Becton Dickinson, San Jose, CA, United States) using Cell Quest, and Win MDI 2.9 softwares was used to acquisition and histograms analysis. The small and large cells were identified by forward (FSC) and side-angle light scatter (SSC) characteristic by flow cytometry. Autofluorescence was assessed by acquiring cells on the flow cytometry without incubating with fluorochrome - labeled antibodies. Surface antigen expression was determined with a variety of directly labeled antibodies according to the supplier’s recommendations.

### Determination of caspase-3 active and cyclin D1 by flow cytometry

The cellular pellet was resuspended in phosphate-buffered solution (PBS) at a concentration 5 × 10^5^ cells/mL and incubated for 1 h at 4°C with 1 μl of specific antibodies: cyclin D1 conjugated with fluorescein isothiocyanate (Abcam, Cambridge, MA, United States) and specific antibody caspase-3 active with phycoerythrin (PE) (Abcam, Cambridge, MA, United States), in absence or presence of an Ac-Asp-Glu-Val-Asp-OH-specific inhibitor (Abcam, Cambridge, MA, United States). After this period the cells were centrifugate at 428 × g for 10 min, washed twice with PBS-cold and fixed with 1% paraformaldehyde. Flow cytometer settings were established using unstaining cells. Cells were gated by forward scatter to eliminated debris. To eliminate the possible autofluorescence of ADSCs, the contribution of unstained cells in the measurement channel was removed. A minimum of 10,000 events was counted for each analysis. Cells were evaluated for cell surface proteins expression using BD Biosciences FacsCalibur flow cytometer (Becton Dickinson, San Jose, CA, United States) using Cell Quest, and Win MDI 2.9 softwares was used to create the histograms.

### Cell cycle phases analysis

The cells were washed with PBS and resuspended in 300 μl 0.03 g/l trypsin, 10 mM Tris (pH 8.0). After 15 min incubation at room temperature, 100ul of the neutralization solution (0.5 g/l trypsin inhibitor, 0.1 g/l RNase A and 1.2 g/l spermine) was added and incubation continued for 15 min. After centrifugation cells were resuspended in 300 μl PBS and fixed by the addition of ice-cold ethanol 70%. Prior to analysis, cells were incubated with 1.8 μg/ml propidium iodide solution (Sigma-Aldrich, St. Louis, MO, United States) and incubated in the dark for 30 min. Flow cytometric analysis was performed using a BD Biosciences FacsCalibur flow cytometer (Becton Dickinson, San Jose, CA, United States) . The DNA content in the cell cycle phases (sub-haploid, G0/G1, S and G2/M) was analyzed by the Cell- Quest software and by the ModFit LT 3.2 software (Becton Dickinson, San Jose, CA, United States).

### Statistical analysis

Numerical values were expressed as mean ± standard deviation. One-way analysis of variance (ANOVA) and Tukey-Kramer multiple-comparisons test were performed to identify differences among measurements of the groups studied and the graphics were obtained by Prism version 5.0 and ModFit version 3.2 softwares. Statistical Significance (p-value) *p < 0.05, **p < 0.01 and ***p < 0.001.
